# Performance of presepsin and procalcitonin predicting culture-proven bacterial infection and 28-day mortality: A cross sectional study

**DOI:** 10.3389/fmed.2022.954114

**Published:** 2022-08-22

**Authors:** Jiho Park, Ji Hyun Yoon, Hyun Kyun Ki, Jae-Hoon Ko, Hee-Won Moon

**Affiliations:** ^1^Division of Infectious Diseases, Department of Medicine, Konkuk University Medical Center, Konkuk University School of Medicine, Seoul, South Korea; ^2^Division of Infectious Diseases, Department of Medicine, Samsung Medical Center, Sungkyunkwan University School of Medicine, Seoul, South Korea; ^3^Department of Laboratory Medicine, Konkuk University Medical Center, Konkuk University School of Medicine, Seoul, South Korea

**Keywords:** presepsin, procalcitonin, bacterial infections, sepsis, prognosis

## Abstract

Presepsin is a highly specific biomarker for diagnosing bacterial infections, but its clinical usefulness is not well validated. A retrospective cross-sectional study was conducted. Among the patients suspected bacterial infection or fulfilled the criteria of systemic inflammatory response syndrome (SIRS) and patients who underwent blood culture, presepsin, procalcitonin (PCT), and C-reactive protein (CRP) at the same time were included. Receiver operating characteristic (ROC) curve analysis and logistic regression were used to compare performance of three biomarkers. A total of 757 patients were enrolled, including 256 patients (33.8%) with culture-proven bacterial infection and 109 patients (14.4%) with bacteremia. The 28-day mortality rate was 8.6%. ROC curve analysis revealed that the area under the curve (AUC) of PCT was higher than that of presepsin for both culture-proven bacterial infection (0.665 and 0.596, respectively; *p* = 0.003) and bacteremia (0.791 and 0.685; *p* < 0.001). In contrast, AUC of PCT for 28-day mortality was slower than presepsin (0.593 and 0.720; *p* = 0.002). In multivariable logistic regression analysis, PCT showed the highest ORs for culture-proven bacterial infection (OR 2.23, 95% CI 1.55–3.19; *p* < 0.001) and for bacteremia (OR 5.18, 95% CI 3.13–8.56; *p* < 0.001), while presepsin showed the highest OR for 28-day mortality (OR 3.31, 95% CI 1.67–6.54; *p* < 0.001). CRP did not show better performance than PCT or presepsin in any of the analyses. PCT showed the best performance predicting culture-proven bacterial infection and bacteremia, while presepsin would rather be useful as a prognostic marker.

## Introduction

Untreated bacterial infections and bacteremia can cause major health problems with a mortality rate as high as 30% (1–3). Early recognition of bacterial infection and administration of empirical antibiotics are essential to improve prognosis for infected patients (4). However, differential diagnosis of bacterial infection from other non-infectious causes of systemic inflammation is often difficult, because fever and leukocytosis have poor sensitivity and specificity in many clinical settings (5, 6). Culture-based approaches remain the gold standard for diagnosis of bacterial infection including bacteremia, but they are time-consuming and results are not available for 12–48 h (6–8). Therefore, recent interest has focused on inflammatory biomarkers for early assessment of bacterial sepsis, including procalcitonin (PCT) and C-reactive protein (CRP) (9), however, these biomarkers could be elevated in non-infectious conditions (10, 11). Presepsin, the soluble fraction of cluster of differentiation 14 (CD14), is suggested as a novel biomarker for bacterial sepsis, which is released into circulation when monocytes are activated after binding with lipopolysaccharides (LPS) and LPS binding protein (12–14). Several studies showed that presepsin is a very good inflammatory biomarker for early diagnosis of sepsis and evaluation of sepsis prognosis (14–24). However, the clinical usefulness of presepsin is still controversial because its superior performance for predicting bacterial infection compared with PCT was observed in relatively small cohorts (14, 17) and most large-scale studies did not include a sufficient number of culture-proven bacterial infections (16, 25–29). To determine the clinical usefulness of presepsin, we evaluated its performance predicting culture-proven bacterial infection among patients with sepsis, in comparison with PCT and CRP.

## Materials and methods

### Patients and study design

The cross sectional study was conducted between January 2020 and October 2020 at Konkuk University Hospital, a 950-bed, community-based tertiary medical center in Seoul, Republic of Korea. We screened the electronic medical records (EMR) of adult patients (≥ 18 years) who were clinically suspected to have bacterial infection and fulfilled the systemic inflammatory response syndrome (SIRS) criteria. Among these patients, patients who underwent blood culture and presepsin at the same time were included. Bacteremia was defined as recovery of any pathogenic bacterial species in one or two sets of blood cultures. Microorganisms commonly considered as contaminants were excluded from the bacteremia group (30). Culture-proven bacterial infection was defined as isolation of pathogens from possible clinical specimens. This study was approved by the Institutional Review Board (IRB) of Konkuk University Medical Center (#2022–04-040) and performed in accordance with the Declaration of Helsinki. Informed consent was waived by the IRB of Konkuk University Medical Center because the EMR was reviewed retrospectively with de-personalized identification numbers.

### Data collection

Data were collected from administrative, pharmaceutical, and laboratory computerized databases maintained by the medical information team at Konkuk University Medical Center. Clinical records were reviewed, and the following information was recorded: age, sex, infection type, blood culture results, Charlson’s weighted index score (CWIs), 28-day mortality, and Quick Sequential Organ Failure Assessment (qSOFA) score. Infection type was clinically established based on clinical symptoms, imaging, and laboratory findings with or without isolation of bacteria from the presumed source (16, 31). The 28-days mortality was defined as death caused by any reasons within 28 days of the presepsin test. qSOFA scores were calculated by checking respiratory rate, Glasgow Coma Scale (GCS) score, systolic blood pressure recorded at the time of obtaining blood for presepsin and culture. Laboratory findings at the same time as presepsin test and blood culture including CRP and PCT were collected. The severity of illness in bacteremia was assessed using the Pitt bacteremia score, which has been validated in several previous studies (32).

### Measurement methods

Plasma presepsin concentrations were measured using an automated chemiluminescent enzyme immunoanalyzer, PATHFAST system (LSI Medience Co., Tokyo, Japan). Presepsin in the sample binds to anti-presepsin antibodies to assemble an immunocomplex with ALP-labeled antibodies and mouse monoclonal antibody-coated magnetic particles. After 10-min incubation with a chemiluminescent substrate, luminescence was generated by the enzyme reaction, a photomultiplier detected, and presepsin concentration was calculated (13). We defined a cut-off value of 314 pg/mL according to the manufacturer’s instructions. Serum PCT levels were measured with an electrochemiluminescence immunoassay (Brahms GmbH, Henningsdorf, Germany) in the Roche Cobas e-System (Roche Diagnostics, Basel, Switzerland). Serum separation tubes were used for CRP. CRP was measured using the latex immunoturbidimetric method with a CRP-Latex X2 (Denka Seiken Co., Tokyo, Japan) on a Toshiba 200FR Autoanalyzer (Toshiba Medical Systems Co., Ltd., Tokyo, Japan) (33).

### Statistical analyses

To compare clinical variables, the Mann-Whitney *U*-test was used for continuous variables, and chi-square and Fisher’s exact tests were used for categorical variables. Age, sex, qSOFA, CWIs (which could be confounding factors), and three biomarkers were included in the multivariable logistic regression model. The area under the receiver-operating-characteristics (ROC) curve estimation was used to evaluate the diagnostic performances of the tested biomarkers and area under the curve (AUC) differences were calculated with the De Long test (34). Optimal cut-off values were derived from ROC curves using the point closest-to-(0,1) corner in the ROC plane which defines the optimal point as the minimizing the distance between the ROC curve and the (0,1) point (35), and sensitivity, specificity, and predictive values were estimated to predict culture-proven bacterial infection with or without bacteremia. The 28-day mortality rates were calculated based on these cut-off values. IBM SPSS Statistics version 20.0 for Windows (IBM, Armonk, NY, United States) was used for all statistical analyses.

## Results

### Study population and microbiology results

Of the 850 patients who were screened for this study, 93 who did not undergo blood culture and presepsin at the same time were excluded. A total of 757 patients were finally included in the study. Culture-proven bacterial infection with or without bacteremia was detected in 256 patients (33.8%). Bacteremia was detected in 109 (14.4%) patients. Gram-negative microorganisms were obtained in 84 samples and Gram-positive organisms in 27 samples. Two or more microorganisms were identified in five patients (4.5%) (Supplementary Table 1). When three biomarkers and Pitt bacteremia score were compared in patients with bacteremia according to microorganism type, no statistically difference was found between patients with Gram-positive, Gram-negative, and poly-microbial infections (Supplementary Table 2).

### Comparison between bacteremia group and non-bacteremia group

Demographic and clinical characteristics of the patients in the bacteremia and non-bacteremia groups are shown in Table 1. There were no differences between the bacteremia and non-bacteremia group regarding age, sex, CWI, and 28-day mortality. Patients with urinary tract infection (33.9 vs. 15.3%, *p* < 0.001) or skin, soft tissue, or bone infection (10.1 vs. 3.5%, *p* < 0.001) were more common in the bacteremia group, whereas the proportion of patients with pneumonia was higher in the non-bacteremia group (46 vs. 7.3%, *p* < 0.001). Presepsin, PCT, and CRP values were higher in the bacteremia group than the non-bacteremia group (*p* < 0.001) (Table 1).

**TABLE 1:** Patient demographic and clinical characteristics.

Variables	Bacteremia (*n* = 109)	Non-bacteremia (*n* = 648)	*p*-value
Sex, male	56 (51.4)	356 (54.9)	0.557
Age (years)	70 (60–79)	72 (61–81)	0.618
**Biomarkers**			
Presepsin, pg/mL	1730.3 ± 1930.0	920.4 ± 1243.9	< 0.001
PCT, ng/mL	16.1 ± 17.2	3.9 ± 10.0	< 0.001
CRP, mg/dL	16.5 ± 11.6	11.1 ± 9.4	< 0.001
**Source of infection**			
Urinary tract	37 (33.9)	99 (15.3)	< 0.001
Pneumonia	8 (7.3)	298 (46)	< 0.001
Intra-abdominal	29 (26.6)	119 (18.4)	0.061
Skin, soft tissue, bone	11 (10.1)	23 (3.5)	0.005
Catheter associated	10 (9.2)	4 (0.6)	< 0.001
Neutropenic fever	13 (11.9)	67 (10.3)	0.741
CNS/deep neck	1 (0.9)	9 (1.4)	1.000
Not specified	0 (0)	29 (4.5)	0.015
Quick SOFA score	0 (0–2)	0 (0–1)	0.001
CWIs	1 (0–2)	1 (0–3)	0.697
28-day mortality	15 (13.8)	50 (7.7)	0.057

Data are expressed as numbers (%) of patients or means ± standard deviations. PCT, procalcitonin; CRP, C-reactive protein; CNS, central nervous system; SOFA, sequential organ failure assessment; CWI, Charlson’s weighted index of comorbidity.

### Diagnostic accuracy of three biomarkers for predicting culture-proven bacterial infection, bacteremia, and 28-day mortality

**FIGURE 1:**
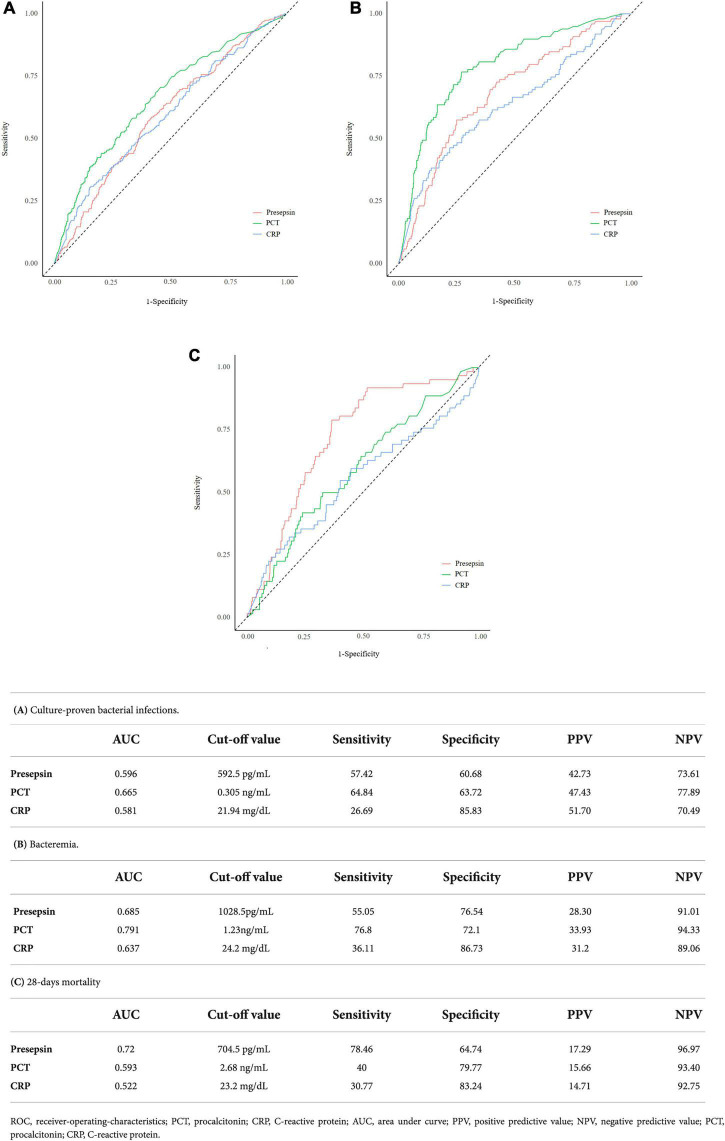
ROC curves for predicting culture proven bacterial infections, bacteremia, and 28-day mortality along with sensitivity, specificity, positive predictive value (PPV), and negative predictive value (NPV) at the best cut-offs for the following parameters: presepsin, PCT, and CRP.

ROC curves for presepsin, PCT, and CRP for predicting culture-proven bacterial infection, bacteremia, and 28-day mortality are shown in Figure 1. The ROC curve analysis for predicting culture-proven bacterial infection with or without bacteremia yielded an AUC value 0.596 (95% CI: 0.551–0.641) for presepsin, 0.665 (95% CI: 0.621–0.709) for PCT, and 0.581 (95% CI: 0.550–0.642) for CRP (Figure 1A). The AUC value of PCT was higher than that of presepsin (*p* = 0.003), while AUC value of presepsin was equal to that of CRP (*p* = 0.996). The cut-off values derived from ROC curves were 592.5 pg/mL for presepsin, 0.305 ng/mL for PCT, and 21.94 mg/dL for CRP. When we used a presepsin cut-off value of 592.5 pg/mL for culture-proven bacterial infection with or without bacteremia, sensitivity, specificity, positive predictive value (PPV), and negative predictive value (NPV) were 57.42, 60.68, 42.73, and 73.61%, respectively.

ROC curve analysis for predicting bacteremia yielded an AUC value of 0.685 (95% CI: 0.628–0.741) for presepsin, 0.791 (95% CI: 0.742–0.840) for PCT, and 0.637 (95% CI: 0.572–0.701) for CRP. The AUC value of PCT was higher than that of presepsin (*p* < 0.001), while AUC value of presepsin was higher than that of CRP (*P* < 0.001) (Figure 1B). The cut-off values derived from ROC curves were 1028.5 pg/mL for presepsin, 1.23 ng/mL for PCT, and 24.2 mg/dL for CRP. When we used a presepsin cut-off value of 1028.5 pg/mL for diagnosing bacteremia, sensitivity, specificity, PPV, and NPV were 55.05, 76.54, 28.30, and 91.01%, respectively.

ROC curve analysis for predicting 28-day mortality yielded an AUC value of 0.72 (95% CI: 0.66–0.781) for presepsin, 0.593 (95% CI: 0.519–0.667) for PCT, and 0.522 (95% CI: 0.467–0.637) for CRP. The AUC value of presepsin was higher than that of PCT (*p* = 0.002) (Figure 1C). The cut-off values derived from ROC curves were 704.5 pg/mL for presepsin, 2.68 ng/mL for PCT, and 23.2 mg/dL for CRP. When we used a presepsin cut-off value of 704.5 pg/mL for predicting 28-day mortality, sensitivity, specificity, PPV, and NPV were 78.46, 64.74, 17.29, and 96.97%, respectively.

### Independent predictors of culture-proven bacterial infection, bacteremia, and 28-day mortality

The cut-off values of three biomarkers derived from the ROC analysis were used for logistic regression analysis (Table 2). PCT (OR = 2.23, 95% CI: 1.55–3.19; *p* < 0.001), CRP (OR 1.62, 95% CI 1.08–2.44; *p* = 0.020), and qSOFA (OR 1.48, 95% CI 1.03–1.98; *p* < 0.001) were found to be the independent predictors of culture-proven bacterial infection, but presepsin was not. Presepsin (OR 2.28, 95% CI 1.41–3.70; *p* < 0.001) and PCT (OR 5.18, 95% CI 3.13–8.56; *p* < 0.001) were found to be the independent predictors of bacteremia, but CRP was not. In contrast, only presepsin among biomarkers was found to be the independent predictors of 28-day mortality (OR 3.31, 95% CI 1.67–6.54; *p* < 0.001), in addition to qSOFA (OR 3.14, 95% CI 2.36–4.18; *p* < 0.001) and CWIs (OR 1.19, 95% CI 1.05–1.34; *p* = 0.006).

**TABLE 2:** Independent factors for predicting culture-proven bacterial infection, bacteremia, and 28-day mortality.

	Variables	Adjusted OR (95% CI)	*p*-value
Culture-proven	Age	1.01 (1.00–1.02)	0.296
Bacterial infection	Sex	1.43 (1.03–1.98)	0.031
	Presepsin ≥ 592.5 pg/mL	1.25 (0.88–1.77)	0.220
	PCT ≥ 0.305 ng/mL	2.23 (1.55–3.19)	< 0.001
	CRP ≥ 21.5 mg/dL	1.62 (1.08–2.44)	0.020
	qSOFA	1.48 (1.03–1.98)	< 0.001
	CWIs	0.98 (0.91–1.06)	0.627
Bacteremia	Age	0.99 (0.97–1.00)	0.120
	Sex	1.31 (0.83–2.06)	0.249
	Presepsin ≥ 1028.5 pg/mL	2.28 (1.41–3.70)	< 0.001
	PCT ≥ 1.23 ng/mL	5.18 (3.13–8.56)	< 0.001
	CRP ≥ 24. 2 mg/dL	1.65 (0.98–2.75)	0.057
	qSOFA	1.21 (0.97–1.53)	0.097
	CWIs	0.94 (0.84–1.04)	0.213
28-day mortality	Age	1.02 (1.00–1.04)	0.094
	Sex	0.91 (0.49–1.66)	0.750
	Presepsin ≥ 704.5 pg/mL	3.31 (1.67–6.54)	< 0.001
	PCT ≥ 2.68 ng/mL	1.02 (0.52–1.98)	0.964
	CRP ≥ 23.2 mg/dL	1.47 (0.74–2.92)	0.273
	qSOFA	3.14 (2.36–4.18)	< 0.001
	CWIs	1.19 (1.05–1.34)	0.006

OR, odds ratio; PCT, procalcitonin; CRP, C-reactive protein; SOFA, sequential organ failure assessment; CWI, Charlson’s weighted index of comorbidity.

## Discussion

Early recognition of bacterial infection including bacteremia is very important for initiating antimicrobial therapy and improving clinical outcomes (27). Biomarkers play an essential role in early identification of bacterial infection, furthermore, bacteremia, sepsis, severe sepsis, and septic shock (10). PCT was regarded as a useful marker for diagnosis of bacterial infection. It could identify patients with sepsis in 96% and septic shock in 98% of cases, which seemed to be superior to SOFA score (36, 37). Moreover, PCT showed better diagnostic and prognostic role in case of gram-negative sepsis and septic shock than gram-positive and fungal sepsis. These superior performance of PCT may make it possible to tailor antimicrobial therapy early (38).

However, as research results showed PCT could be elevated in non-infectious conditions such as postoperative settings, cardiogenic shock, trauma, burn, acute pancreatitis and acute graft-vs.-host disease, efforts were made to find another ideal biomarker due to these limitations (10, 11). Presepsin, a new diagnostic biomarker for sepsis, is highly specific for diagnosing bacterial infections because its production is associated with bacterial phagocytosis and cleavage of microorganisms by lysosomal enzymes. It was proven to be secreted from granulocytes by infectious stimuli in an animal study (39).

Therefore, we evaluated the usefulness of presepsin to predict diagnosis of culture-proven bacterial infection and bacteremia in adult patients relative to other biomarkers. We retrospectively collected measurement the level of three biomarkers, presepsin, PCT and CRP in patients with suspected different infectious conditions on the day of occurrence of it and also collected final culture results. All three biomarkers and qSOFA scores were associated with bacteremia in univariable analyses. Among pneumonia cases, eight patients had bacteremia (7.3%) while 298 patients did not (46%). It may be that, with pneumonia, it is only possible to diagnose the causative pathogen in 30–40% of cases using conventional diagnostic methods (40).

ROC curve analysis demonstrated that PCT was superior to presepsin and CRP as a diagnostic biomarker, and it had higher sensitivity and negative predictive value for predicting culture-proven bacterial infection and bacteremia. Numerous studies showed that presepsin is a good inflammatory marker for sepsis, wherein it showed better sensitivity, specificity, and diagnostic accuracy than PCT. However, only a few studies with a small number of patients focused on the role of presepsin for predicting bacterial infection including bacteremia and each study yielded conflicting results (25–28). Leli et al. conducted a study with 92 patients with suspected sepsis. Bacteremia was confirmed in 32 of 92 patients, and they showed that both presepsin and PCT had good diagnostic accuracy in predicting bacteremia, superior to CRP (25). Romualdo et al. produced similar results, wherein bacteremia was confirmed in 37 of 226 patients with SIRS and presepsin and PCT showed similar potential to differentiate between SIRS patients with and without bacteremia. The AUC value of presepsin for predicting bacteremia was higher than PCT (26). Imai et al. conducted a prospective study with 46 elderly patients and the AUC values were not different among presepsin and PCT (27). However, these studies evaluated the utility of presepsin for predicting bacteremia with a relatively small population. In contrast, our study evaluated 757 patients all with suspected infection from any origin. Among them, 256 patients had culture-proven bacterial infection, and 109 of those infected patients were confirmed to have bacteremia.

The optimal cut-off value for presepsin for diagnosing bacteremia in our study was 1028.5 pg/mL, relatively higher than other studies (14–16, 23). However, as with most prospective studies, patients who were suspected to have sepsis or septic shock at the time of admission were included, patients who had non-infectious etiologies that manifested like sepsis or septic shock or who had no bacterial infections may have been included. In a bacteremia study, the authors suggested an optimal presepsin cut-off value of 729 pg/mL, but this study only included 37 bacteremic patients (26). The retrospective cross sectional study design, characteristics of the single-center study, and patient diversity including hospitalized patients as well as hospitalization through the emergency department may have influenced the high cut-off value of presepsin.

Whether presepsin can distinguish between Gram-positive and Gram-negative bacterial infections is still controversial (21, 23, 41). It has been hypothesized that presepsin levels can differentiate type of bacterial origin from the fact that presepsin is a receptor of LPS, which is one of the components of Gram-negative bacteria (23). Although, there was a difference in presepsin levels between Gram-positive and Gram-negative bacterial infections, the difference was not statistically meaningful (947.5 vs. 1232.5, *P* = 0.705).

Some studies concluded that presepsin could be used to assess the severity of inflammatory disease without infection or viral disease. These studies showed that inappropriate monocyte or neutrophil activation due to systemic lupus erythematous flare-up had induced elevation of presepsin levels. These results also suggested that presepsin production was influenced by monocyte phagocytosis from a neutrophil extracellular trap (42, 43). Presepsin has also been suggested as a predictive biomarker of severity in COVID-19 infections. Severe COVID-19 infections could cause a systemic inflammatory reaction combined with elevated cytokines, such as monocyte chemoattractant protein 1 and macrophage inflammatory protein 1a. These cytokines may stimulate presepsin production (44, 45). Combining the results of previous studies and the result of our study, presepsin may be a specific marker for clinical situations involving monocyte activation rather than being specific to bacterial infection.

Taken together, presepsin could be a useful prognostic factor for 28-day-mortality rather than a predictor of bacterial infection.

This study had several limitations. First, there is a potential selection bias because the suspicion of bacterial infection was made freely by physicians. Second, we did not classify according to time of blood sample collection. It is known that presepsin specifically increases within a few hours of clinically suspected sepsis (15). However, because this was a cross-sectional study, it was not possible to compare each biomarker serially over time in clinical situations of suspected bacterial infection with or without bacteremia. Additional validation through cohort study is required. Third, we did not exclude patients with acute kidney injury (AKI) or end-stage kidney disease (ESRD), knowing that renal function could falsely increase presepsin levels. In our study, there were only 40 patients with AKI or ESRD, and additional evaluations were not performed.

In conclusion, among the evaluated biomarkers, PCT showed best performance predicting culture-proven bacterial infection and bacteremia while presepsin was more useful as a prognostic marker. Further studies are necessary to better understand the role of presepsin in various clinical settings, such as viral infection, fungal infection, and non-infectious inflammatory conditions.

## Data availability statement

The original contributions presented in the study are included in the article/Supplementary material, further inquiries can be directed to the corresponding author/s.

## Ethics statement

The studies involving human participants were reviewed and approved by the Institutional Review Board (IRB) of Konkuk University Medical Center (#2022–04-040). Written informed consent for participation was not required for this study in accordance with the national legislation and the institutional requirements.

## Author contributions

JP, J-HK, H-WM, JY, and HK: conceptualization. JP and H-WM: investigation. H-WM: laboratory work and methodology. JP: supervision. JP, J-HK, and H-WM: writing—review and editing. All authors have read and agreed to the publication of this manuscript.
