# Supplementary material for: Performance of presepsin and procalcitonin predicting culture-proven bacterial infection and 28-day mortality: A cross sectional study
Source: Front Med (Lausanne). 2022 Aug 22;9:954114. doi: 10.3389/fmed.2022.954114 (PMC9441687; doi:10.3389/fmed.2022.954114)
Supplement: Supplementary file 1 [file Table_1.pdf]

SUPPLEMENTAL DATA

Supplementary Table 1. Pathogens detected by culture methods, responsible for mono-microbial or poly-microbial including blood-stream infections, in 256 of 757 patients

Microorganism	Culture-proven bacterial infection† (n=256)	Bacteremia† (n=109)
**Gram positive bacteria**	73	27
Staphylococcus aureus	44	13
*Coagulase-negative Staphylococcus**	3	2
Streptococcus pneumoniae	5	2
Viridans streptococci	4	3
Clostridium spp.	2	2
Enterococcus spp.	14	4
Eggerthella spp.	1	1
**Gram negative bacteria**	226	84
Escherichia coli	101	44
Klebsiella pneumoniae	41	21
Klebsiella aerogenes	3	2
Klebsiella oxytoca	2	0
Enterobacter cloacae	7	2
Serratia marcescens	2	1
Proteus mirabilis	12	3
Moraxella morganii	4	0
Acinetobacter baumannii	8	0
Pseudomonas aeruginosa	32	2
Stenotrophomonas maltophilia	4	2
Aeromonas hydrophilia	1	0
Bacteroides fragilis	3	3
Parvimonas micra	1	1
Providencia rettgeri	1	1
Gardnerella vaginalis	1	0
**Polymicrobial identification**	38	5

*Isolated from more than two consecutive blood cultures and considered to be true pathogen by the attending physicians.

†No. of patients does not equal the no. of pathogens because some patients had multiple pathogens detected.

Supplementary Table 2. Comparison of biomarkers in patients with bacteremia according to microorganism type

	Type of microorganism			
	Gram positive (n=26)	Gram negative (n=78)	Polymicrobial (n=5)	***P*-value**
Presepsin (pg/mL)	947.5 (525.2–1682.0)	1232.5 (556.8–2301.2)	1154.0 (445–1671.0)	0.705
PCT (ng/mL)	7.7 (0.4–16.0)	10.7 (1.6–31.1)	47.8 (23.9–48.5)	0.252
CRP (mg/dl)	16 (4.8–28.3)	16 (5.6–29.0)	10.6 (0.8–11.9)	0.601
Pitt bacteremia score ≥4	2 (7.7)	9 (11.5)	1 (20)	0.506

Abbreviations: PCT, procalcitonin; CRP, C-reactive protein
